# Multiplex PCR for detection of plasmid-mediated colistin resistance determinants, *mcr-1, mcr-2, mcr-3, mcr-4* and *mcr-5* for surveillance purposes

**DOI:** 10.2807/1560-7917.ES.2018.23.6.17-00672

**Published:** 2018-02-08

**Authors:** Ana Rita Rebelo, Valeria Bortolaia, Jette S Kjeldgaard, Susanne K Pedersen, Pimlapas Leekitcharoenphon, Inge M Hansen, Beatriz Guerra, Burkhard Malorny, Maria Borowiak, Jens Andre Hammerl, Antonio Battisti, Alessia Franco, Patricia Alba, Agnes Perrin-Guyomard, Sophie A Granier, Cristina De Frutos Escobar, Surbhi Malhotra-Kumar, Laura Villa, Alessandra Carattoli, Rene S Hendriksen

**Affiliations:** 1 National Food Institute, Technical University of Denmark, WHO Collaborating Center for Antimicrobial Resistance in Food borne Pathogens and European Union Reference Laboratory for Antimicrobial Resistance, Kongens Lyngby, Denmark; 2 European Food Safety Authority, Parma, Italy; 3 German Federal Institute for Risk Assessment, Berlin, Germany; 4 National Reference Laboratory for antimicrobial resistance, Istituto Zooprofilattico Sperimentale del Lazio e della Toscana, Rome, Italy; 5 Anses, Fougères Laboratory, Fougères, France; 6 Université Paris-Est, Anses, Laboratory for Food Safety, Maisons-Alfort, France; 7 Laboratorio Central de Veterinaria, (LCV Algete), Madrid, Spain; 8 Laboratory of Medical Microbiology, Vaccine & Infectious Disease Institute, University of Antwerp, Wilrijk, Belgium; 9 Department of Infectious Diseases, Istituto Superiore di Sanità, Rome, Italy

**Keywords:** polymerase chain reaction, multiplex, colistin, antimicrobial resistance, transferable resistance, polymyxin E, *mcr*, surveillance, *mcr-4.3*

## Abstract

Plasmid-mediated colistin resistance mechanisms have been identified worldwide in the past years. A multiplex polymerase chain reaction (PCR) protocol for detection of all currently known transferable colistin resistance genes (*mcr-1* to *mcr-5*, and variants) in *Enterobacteriaceae* was developed for surveillance or research purposes. **Methods:** We designed four new primer pairs to amplify *mcr-1*, *mcr-2*, *mcr-3* and *mcr-4* gene products and used the originally described primers for *mcr-5* to obtain a stepwise separation of ca 200 bp between amplicons. The primer pairs and amplification conditions allow for single or multiple detection of all currently described *mcr* genes and their variants present in *Enterobacteriaceae*. The protocol was validated testing 49 European *Escherichia coli* and *Salmonella* isolates of animal origin. **Results:** Multiplex PCR results in bovine and porcine isolates from Spain, Germany, France and Italy showed full concordance with whole genome sequence data. The method was able to detect *mcr-1, mcr-3* and *mcr-4* as singletons or in different combinations as they were present in the test isolates. One new *mcr-4* variant, *mcr-4.3*, was also identified. **Conclusions:** This method allows rapid identification of *mcr*-positive bacteria and overcomes the challenges of phenotypic detection of colistin resistance. The multiplex PCR should be particularly interesting in settings or laboratories with limited resources for performing genetic analysis as it provides information on the mechanism of colistin resistance without requiring genome sequencing.

## Introduction

Colistin belongs to the antimicrobial class designated polymyxins which originates from the organism *Paenibacillus polymyxa*. This class consists of polymyxins A, B, C, D and E, of which only colistin (polymyxin E) and polymyxin B are used in clinical practice [1]. Colistin has been widely used in veterinary medicine in Asian, European and North American countries but human use was restricted due to its neuro- and nephrotoxicity [2-4]. In the past decade, emergence of multidrug-resistant Gram-negative bacteria (such as *Enterobacteriaceae*, *Pseudomonas aeruginosa* and *Acinetobacter baumannii*) led to an increase of colistin administration as a last resort antibiotic for human infections [5]. Prior to the detection of the plasmid-mediated resistance gene *mcr*-*1*, resistance to colistin in *Enterobacteriaceae* had only involved chromosomal mutations, including mutations in the *pmrA/pmrB* two-component system that regulate synthesis and structure of the lipopolysaccharide [6].

Global surveillance of plasmid-mediated colistin resistance is hindered by the technical difficulties of phenotypic tests owed to particular interactions of the drug with reagents or materials and resulting from its specific molecular structure. The main problems associated with phenotypic testing are the (i) irregular diffusion of colistin in agar media, (ii) interaction with cations in media and (iii) possible adsorption of the antibiotic to certain laboratory materials, which make the agreement of results between replicated testing and between laboratories difficult. Disk diffusion, agar dilution and Etest strips methods cannot be used to perform colistin susceptibility testing, and there is yet not enough information available regarding automated methods to reach a significant conclusion about their sensitivity [1,7]. Thus to date, only determination of minimum inhibitory concentration (MIC) by broth microdilution is recommended in technical guidance documents by the European Committee on Antimicrobial Susceptibility Testing (EUCAST) and the Clinical and Laboratory Standards Institute (CLSI) [8,9].

The first plasmid-mediated colistin resistance gene was identified in an *Escherichia coli* strain from a pig in China and also found to be present in other *Escherichia coli* and *Klebsiella pneumoniae* isolates in samples from both animal and human origin, collected between 2011 and 2014 [4]. The *mcr-1* gene (KP347127) encodes a phosphoetanolamine transferase that alters the lipid A in the lipopolysaccharide of the bacterial outer membrane. This reduces the attachment of colistin and therefore prevents cell lysis. Although this mechanism has only recently been discovered there are reports of isolates recovered in 1980 harbouring the *mcr-1* gene, but most cases refer to isolates collected since 2009 [10]. After the first detection, additional four *mcr* genes were described: *mcr-2* (LT598652) was found in *E. coli* from animal sources in Belgium [11], *mcr-3* (KY924928) in *E. coli* from pigs in China [12], *mcr-4* (MF543359) in *E*. *coli* and *Salmonella enterica* serovar Typhimurium from pigs in Italy, Spain and Belgium [13] and *mcr-5* (KY807921) in *Salmonella* Paratyphi B dTa + from poultry in Germany [14]. Variants of *mcr-1,* *mcr-3* and *mcr-4* [15-18] as well as co-occurrence of *mcr-1* and *mcr-3* have been observed in *Enterobacteriaceae* [19,20].

Even though *mcr* genes have been more prevalent in animal and food isolates than in human strains, multiple cases of human infection by strains harbouring *mcr-1*, *mcr-3* or *mcr-4 *have been observed [15,18,21]. Worldwide, over 100 *Enterobacteriaceae* strains harbouring *mcr* have been recovered from human hosts, mostly *E. coli* strains, in patients originating from, or who had recently travelled to, Asian countries [15]. This includes the first *mcr-1* description by Liu et al. who identified 16 *mcr-1*-positive *E. coli* and *K. pneumoniae* clinical strains [4]. More recently, *mcr-1 *and *mcr-1.*2 were described in one *E. coli* and one *K. pneumoniae* strain from infected humans in Denmark [22] and Italy [23], respectively. Three additional *E. coli* strains carrying the *mcr-1* gene were found in faecal samples of Dutch travellers [24] and a study of pilgrims travelling to Mecca revealed the gene in 10 *E. coli* and one *K. pneumoniae* strains [25]. These findings represent a snapshot of the cases so far described of *mcr-1* in *Enterobacteriaceae* of human origin and illustrate the global spread of bacteria carrying *mcr-1*. In Denmark, one *E. coli* and 10 *Salmonella* clinical strains collected between 2009 and 2017 were found to harbour *mcr-3*, six of which were present in people reporting previous travel to Thailand or Vietnam [17,19]. In Italy, *mcr-4*-positive *Salmonella* strains have been isolated from two humans with gastroenteritis [18].

The discovery of plasmid-mediated colistin resistance has raised international concern as the spread of this resistance might hamper the efficacy of colistin in humans, although the full clinical relevance has not yet been determined. Plasmid-mediated resistance genes have the potential to be further transmitted and spread globally, including to and within regions with already high levels of antimicrobial resistance where multidrug-resistant bacteria represent a public health concern. This can lead to the emergence of colistin-resistance in already multidrug-resistant bacteria, for which colistin represents a last-resort antibiotic, and compromise antibiotic treatment. Thus, human infections for which therapeutic options have been depleted could occur in nosocomial settings.

Considering (i) the global concerns, (ii) the unreliability of most phenotypic methods for colistin susceptibility testing for surveillance purposes, and (iii) the limited availability of whole genome sequencing (WGS) technology in some settings or laboratories with a lack of adequate resources, we aimed to develop and validate a method that enables screening of relevant isolates in a cheap, rapid and efficient way to identify those possibly harbouring plasmid-mediated colistin resistance genes and meriting further characterisation. Here, we describe a multiplex PCR protocol that detects *mcr-1, mcr-2, mcr-3, mcr-4* and *mcr-5* genes rapidly and reliably for surveillance purposes and epidemiological research, particularly in food, animal and environmental samples.

## Methods

### The multiplex PCR method development

To allow fast and simultaneous detection of all the currently described *mcr* genes and their variants and improve visualisation by gel electrophoresis, we focused on conventional block multiplex PCR technique. It was chosen because of its simplicity that enables it to be used in all laboratories and settings with limited laboratory resources, limited or no access to WGS, and in situations where a rapid answer is desired.

The primers for *mcr-5* were those described in the original study whereas the primers for *mcr-1* (320 bp), *mcr-2* (715 bp), *mcr-3* (929 bp) and *mcr-4* (1116 bp) were designed in this study (Table 1) in order to obtain sequential amplicons with a stepwise size separation of ca 200 bp and allow easy visualisation of bands on agarose gels.

**Table 1:** Primers and positive control strains used for multiplex PCR for detection of *mcr-1*, *mcr-2*, *mcr-3*, *mcr-4* and *mcr-5* genes

Primer name	Sequence (5’-3’)	Target gene	Size (bp)	Positive control strain	Reference
*mcr1*_320bp_fw	AGTCCGTTTGTTCTTGTGGC	*mcr-1*	320	*Escherichia coli* 2012–60–1176–27 [22]	This study
*mcr1_*320bp*_*rev	AGATCCTTGGTCTCGGCTTG				
*mcr2*_700bp_fw	CAAGTGTGTTGGTCGCAGTT	*mcr-2*	715	*E. coli* KP37 [11]	This study
*mcr2*_700bp_rev	TCTAGCCCGACAAGCATACC				
*mcr3*_900bp_fw	AAATAAAAATTGTTCCGCTTATG	*mcr-3*	929	*E. coli* 2013-SQ352	This study
*mcr3*_900bp_rev	AATGGAGATCCCCGTTTTT				
*mcr4*_1100bp_fw	TCACTTTCATCACTGCGTTG	*mcr-4*	1,116	*E. coli DH5α* [13]	This study
*mcr4*_1100bp_rev	TTGGTCCATGACTACCAATG				
*MCR5*_fw	ATGCGGTTGTCTGCATTTATC	*mcr-5*	1,644	*Salmonella* 13-SA01718 [14]	[14]
*MCR5*_rev	TCATTGTGGTTGTCCTTTTCTG				

As determined by the Basic Local Alignment Search Tool (BLAST) at the National Center for Biotechnology Information (NCBI), these primers amplify all *mcr* variants detected in *Enterobacteriaceae* to date: *mcr-1.2* (KX236309), *mcr-1.3* (KU934208), *mcr-1.4* (KY041856), *mcr-1.5* (KY283125), *mcr-1.6* (KY352406), *mcr-1.7* (KY488488), *mcr-1.8* (KY683842), *mcr-1.9* (KY964067.1), *mcr-1.11* (KY853650.1), *mcr-1.12* (LC337668.1), *mcr-1.xx* (a new *mcr-1* variant, submitted for publication but not yet definitely named), *mcr-3.2* (NPZH01000177.1), *mcr-3.4* (FLXA01000011.1), *mcr-3.5* (MF463699.1)*, mcr-3.10* (MG214533.1), *mcr-3.11* (MG489958.1), *mcr-4.2* (MG581979) and *mcr-5.xx* (a new *mcr-5* variant, submitted for publication but not yet definitely named). The remaining variants known so far, *mcr-1.10* (MF176238.1)*, mcr-2.2* (NG_055496.1)*, mcr-3.3* (MF495680.1)*, mcr-3.6* (MF598076.1)*, mcr-3.7* (MF598077.1)*, mcr-3.8* (MF598079.1) and *mcr-3.9* (MF598080.1) have not been reported in *Enterobacteriaceae*, having only been found in *Moraxella* spp. and *Aeromonas* spp.. A full overview of *mcr* genes and their variants known to date can be found in the Supplement. BLAST analysis also revealed neither interaction between primer pairs nor unspecific binding to other genes.

The original reference strains for *mcr-2* (*E. coli* KP37) and *mcr-5* (*Salmonella* Paratyphi B dTa + 13-SA01718), and strains available at the Technical University of Denmark in Lyngby for *mcr-1* (*E. coli* 2012–60–1176–27), *mcr-3* (*E. coli* 2013-SQ352) and *mcr-4* (*E. coli DH5α* with the entire *mcr-4* gene cloned in pCR2 vector) were used as positive controls in the multiplex PCR (Table 1). DNA templates were obtained from overnight agar cultures by thermal cell lysis. Each PCR reaction consisted of 12.5 µL DreamTaq Green PCR Master Mix (Thermo Fisher Scientific, Waltham, Massachusetts, United States), 5.5 µL of nuclease-free water, 0.5 µL of each of the 10 primers solutions (10 µM), and 2 µL DNA lysate. Running conditions were: 1 cycle of denaturation at 94 °C for 15 min, followed by 25 cycles of denaturation at 94 °C for 30 s, annealing at 58 °C for 90 s and elongation at 72 °C for 60 s, and a final cycle of elongation at 72 °C for 10 min. The amplification was visualised by electrophoresis using 1.5% agarose gel at 130V followed by staining in ethidium-bromide.

An internal amplification control was not used due to the incompatibility with DreamTaq Green PCR Master Mix, which contains a DNA polymerase synthesised in *E. coli* and thus yields amplicons when using 16S rRNA primers.

### Test isolates for validation of the multiplex PCR

To validate the multiplex PCR, 49 isolates from four European countries were used. These isolates were collected within the framework of the European Monitoring of Antimicrobial Resistance pertaining to the Commission Implementing Decision 2013/652/EU [26]. They were analysed at the European Union Reference Laboratory for Antimicrobial Resistance (EURL-AR) in the context of animal health and food safety, in accordance with the EURL-AR/European Food Safety Authority (EFSA) confirmatory testing, that consists of MIC determination by broth microdilution and WGS by Illumina Hi-Seq (Illumina, Inc., San Diego, California, United States) (European Nucleotide Archive project PRJEB21546). The confirmatory testing represents a random snapshot of submitted unusual phenotypes within each participating country based on selective criteria from EFSA.

## Results

Isolates collected in 2016 from Spain (n = 19), Germany (n = 16), France (n = 10) and Italy (n = 4) were selected for this study due to the diverse colistin resistance profiles found by WGS in these countries. Forty-two isolates were *E. coli* from porcine (n = 20) and bovine (n = 22) sources (including caecum and meat samples) and seven isolates were *Salmonella* spp. from porcine (n = 5) and bovine (n = 2) sources.

The multiplex PCR protocol specifically amplified the fragments of the five *mcr* genes. Amplicon size ranged from 320 bp (*mcr-1*) to 1,644 bp (*mcr-5*) (Figure).

**Figure:**
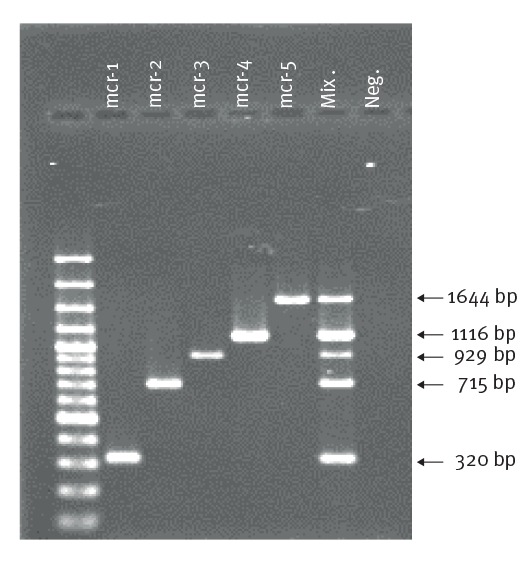
Multiplex PCR for detection of *mcr-1, mcr-2, mcr-3, mcr-4* and *mcr-5*, European Union Reference Laboratory for Antimicrobial Resistance (EURL-AR) in the context of animal health and food safety, 2017

Visualisation of the bands on an agarose (1.5%) gel for the five positive control strains. GeneRuler 100 bp Plus DNA Ladder (Thermo Fisher Scientific, Waltham, Massachusetts, United States) was used as molecular size marker and the size of each amplicon is indicated at the side. Mix corresponds to results obtained with a 5 µL mixture of DNA from all five control strains. Neg. corresponds to the blank negative control.

No interference between primer pairs and no unspecific products were observed. Analysing the validation set, the method detected *mcr-1*, *mcr-3* and *mcr-4* as singletons and in different combinations (Table 2).

**Table 2:** Multiplex PCR for detection of *mcr-1*, *mcr-2*, *mcr-3*, *mcr-4* and *mcr-5* compared with WGS results, applied to a collection of *Escherichia coli* and *Salmonella* spp. isolates from food animals and food, European Union Reference Laboratory for Antimicrobial Resistance in the context of animal health and food safety, Spain, Germany, France, Italy, 2016 (n = 49)

Isolate ID	Species	Origin	Source	Colistin MIC	Colistin resistance by PCR	Colistin resistance by WGS	MLST^a^	Plasmid content^a^	Antimicrobial resistance genes and chromosomal mutations^a^
**Spain (n=19)**									
150721	*E. coli*	Calf	Meat	≤ 1	*-*	*-*	ST-1488	FII, FIB(AP001918), I1, Q1, Col(MG828)	*bla*_CTX-M-32_, *bla*_TEM-1B_, *dfrA1, erm*(B), *mph*(B), *strA, strB, sul1, tet*(A), *tet*(B), *gyrA* S83L, *gyrA* D87N, *parC* S80I
151570	*Salmonella* Kedougou	Pig	Carcass	4	*mcr-4*	*mcr-4.3* ^b^ (V236F)	ST-1543	ColE10, ColpVC, ColRNAI	*dfrA14, strB, sul2*
151885	*E. coli*	Pig	Meat	4	*mcr-1*	*mcr-1.xx*	ST-533	FIA(HI), HI1A, HI1B(R27)	*aph(3')-Ic, bla*_CTX-M-32_, *dfrA1, floR, mph*(E), *msr*(E), *strA, strB, sul1, tet*(B), *gyrA* S83L, *gyrA* D87N, *parC* S80I
151916	*Salmonella* Rissen	Pig	Carcass	≤ 1	-	*-*	ST-469	ColRNAI, R	*aadA1, aadA2, bla*_TEM-1B_, *dfrA12, mph*(A), *sul1, tet*(A)
152169	*E. coli*	Pig	Meat	4	*mcr-1*	*mcr-1*	ST-58	FII(pCoo), FIB(AP001918), I1, N, R, X4, Col156, Col8282	*aac(3)-IId, aadA2, bla*_CTX-M-1_, *bla*_TEM-1B_, *dfrA12, mph*(A), *qnrS1, sul1, tet*(A)
ZTA15/00213–1EB1	*E. coli*	Calf	Caecum	8	*-*	*pmrB* D283G/Y358N	ST-641	FII, I1, X1, p0111, Col156, ColRNAI	*aadA1, aadA2, bla*_SHV-12_, *bla*_TEM-1A_, *cmlA1, erm*(B), *sul1, sul3, tet*(M), *gyrA* S83L, *gyrA* D87N, *parC* S80I
ZTA15/00420EB1	*E. coli*	Pig	Caecum	8	*mcr-1* and *mcr-4*	*mcr-1* and *mcr-4* (441delT)	ST-10	FII(pRSB107), HI2, HI2A, I1, Q1, X4, TrfA, Col(MG828), Col156, ColE10, ColRNAI	*aac(3)-IVa, aadA1, aph(3')-Ia, aph(4)-Ia, bla*_CTX-M-14_, *bla*_TEM-1B_, *dfrA1, mph*(B), *strA, strB, sul1, sul2, tet*(A), *tet*(M), *gyrA* S83L
ZTA15/00685EB1	*E. coli*	Pig	Caecum	2	*mcr-1*	*mcr-1*	ST-457	FIB(AP001918), FIC(FII), FII, I1, R, X4, Col(MG828), ColRNAI	*aac(3)-IVa, aadA1, aadA2, aph(3')-Ia, aph(4)-Ia, bla*_CTX-M-27_, *catA2, cmlA1, dfrA12, erm*(B), *floR, mph*(A), *strA, strB, sul2, sul3, tet*(M), *gyrA* S83L, *gyrA* D87N, *parC* S80I
ZTA15/01045EB1	*E. coli*	Pig	Caecum	≤ 1	*-*	*-*	ST-156	FIA, FIB(AP001918), FII, I1, X1, Col(MG828), ColRNAI	*bla*_CTX-M-14_, *tet*(A), *gyrA* S83L
ZTA15/01169–1EB1	*E. coli*	Calf	Caecum	8	*mcr-1* and *mcr-3*	*mcr-1* and *mcr-3.2*	ST-533	FIB(AP001918), FIC(FII), HI2, HI2A, I1, Y, TrfA, Col156, ColRNAI	*aac(3)-IId, aadA2, bla*_CTX-M-55_, *bla*_TEM-1A_, *dfrA1, floR, mph*(A), *mph*(B), *strA, strB, sul1, sul3, tet*(A), *tet*(M), *gyrA* S83L, *gyrA* D87N, *parC* S80I
ZTA15/00944–1EB1	*E. coli*	Calf	Caecum	≤ 1	*-*	*-*	ST-1721	FIB(pHCM2), R, X1	*aac(3)-IVa, aph(4)-Ia, bla*_CTX-M-1_, *bla*_TEM-1B_, *floR, mph*(A), *qnrS1, strA, sul2, tet*(B)
ZTA15/00877EB1	*E. coli*	Pig	Caecum	≤ 1	*-*	*-*	ST-654	FIB(K), X1, Y	*aadA1, aadA2, bla*_TEM-1B_, *bla*_SHV-12_, *cmlA1, floR, qnrS1, sul3, tet*(A)
ZTA15/00421EB1	*E. coli*	Pig	Caecum	≤ 1	-	*-*	ST-410	FII, FIA, FIB(AP001918), ColRNAI	*aac(6')-Ib-cr, aadA1, aadB, bla*_CTX-M-15_, *bla*_OXA-1_, *catB4, floR, sul1, sul2, tet*(A), *tet*(M), *gyrA* S83L, *gyrA* D87N, *parC* S80I
ZTA15/00380EB1	*E. coli*	Pig	Caecum	≤ 1	-	-	ST-533	FII(p14), FIA, FIB(AP001918), I1, Y, ColRNAI	*aac(3)-IVa, aadA1, aph(4)-Ia, bla*_CTX-M-27_, *dfrA1, floR, strA, strB, sul1, sul2, tet*(A), *gyrA* S83L, *gyrA* D87N, *parC* S80I
ZTA15/00747EB1	*E. coli*	Pig	Caecum	≤ 1	-	-	ST-75	FII, FIA, FIB(AP001918), I1, Col8282, ColRNAI	*bla*_CTX-M-1_, *bla*_TEM-1B_, *mph(A), tet*(A)
ZTA15/00919EB1	*E. coli*	Pig	Caecum	≤ 1	-	-	ST-453	FII, FIB(AP001918), B/O/K/Z, X1, Q1	*aadA2, bla*_CTX-M-1_, *bla*_TEM-1B_, *dfrA5, qnrS1, strA, strB, sul2, gyrA* S83L, *gyrA* D87N, *parC* S80I
ZTA15/01816–1EB1	*E. coli*	Calf	Caecum	≤ 1	-	-	ST-58	FII(pCoo), FIA(HI1), FIB(pB171), B/O/K/Z, ColRNAI	*bla*_CTX-M-14_, *strA, strB, sul2, tet*(B)
ZTA15/00170EB1	*E. coli*	Pig	Caecum	≤ 1	-	-	ST-48	FII, FIB(AP001918), I1, Q1, X1	*aadA1, bla*_CTX-M-14_, *bla*_TEM-1B_, *dfrA1, mph*(B), *qnrS1, strA, strB, sul1, sul2, tet*(A)
ZTA15/02174–1EB1	*E. coli*	Calf	Caecum	≤ 1	-	-	ST-10	FIB(AP001918), FIC(FII), I1, Col156, ColRNAI	*aadA1, aph(3')-Ic, bla*_CTX-M-1_, *bla*_OXA-1_, *bla*_TEM-1B_, *catA1, floR, strA, strB, sul1, sul2, tet*(B), *gyrA* S83L, *gyrA* D87N, *parC* S80I, *parE* S458A
**Germany (n=16)**									
15-AB01393_0	*E.coli*	Calf	Meat	≤ 1	*-*	-	ST-58	FII, FIB, Q1, X1	*bla*_CTX-M-1_, *mph*(A), *strA, strB, sul2*
15-AB01299_0	*E.coli*	Pig	Caecum	4	*mcr-4*	*mcr-4.2*	ST-410	FII, FII(pRSB107), FIA, FIB(AP001918), X1, ColE10, ColRNAI	*aadA1, aadB, bla*_CTX-M-15_, *floR, sul1, sul2, tet*(A), *gyrA* S83L, *gyrA* D87N, *parC* S80I, *parE* S458A
15-AB02002_0	*E.coli*	Calf	Caecum	8	*mcr-1*	*mcr-1*	ST-10	FII, FIB(AP001918), HI2, HI2A, I1, Q1, X1, TrfA, Col156, ColE10, ColRNAI	*aadA1, aadA2, aac(3)-IIa, aph(3')-Ic, bla*_TEM-1A,_ *catA1, cmlA1, dfrA1, strA, strB, sul1, sul2, sul3, tet*(A), *tet*(B)
15-AB01235_0	*E.coli*	Pig	Meat	≤ 1	*-*	*-*	ST-1952	FII, I1, Q1, Col(MG828)	*aadA1, bla*_CTX-M-1_, *bla*_TEM-1B_, *dfrA1, floR, mph*(A), *strA, strB, sul1, sul2, tet*(A), *gyrA* S83L
15-AB01370_0	*E.coli*	Pig	Caecum	≤ 1	*-*	*-*	ST-542	I1	*aadA1, aadA5, bla*_CTX-M-1_, *dfrA17, sul2*
15-AB01894_0	*E.coli*	Pig	Caecum	≤ 1	*-*	*-*	ST-88	FII(pCoo), FIB(AP001918), FIC(FII), I1, IncX1, Col(MG828), Col8282, ColRNAI	*aadA1, aadA5, bla*_CTX-M-1_, *dfrA1, dfrA17, sul1, sul2*
15-AB01509_0	*E.coli*	Calf	Caecum	≤ 1	*-*	*-*	ST-694	FII, FII(pRSB107), FIA, FIB(AP001918), Col156, ColRNAI, B/O/K/Z	*aac(3)-IIa, aac(6')-Ib-cr, aadA5, bla*_CTX-M-15_, *dfrA17, mph*(A), *strA, strB, sul1, sul2, tet*(B), *gyrA* S83L, *gyrA* D87N, *parC* S80I, *parE* S458A
15-AB01308_0	*E.coli*	Calf	Caecum	≤ 1	*-*	*-*	ST-224	Y	*bla*_CTX-M-15_, *bla*_TEM-1B_, *dfrA14, qnrS1, strA, strB, sul2, tet*(A), *gyrA S83L, gyrA D87N, parC S80I, parE S458A*
15-AB01312_0	*E.coli*	Calf	Caecum	≤ 1	*-*	*-*	ST-641	FII, Q1	*aac(3)-IVa, aph(4)-Ia, bla*_CTX-M-1_, *bla*_TEM-1B_, *dfrA7, mph*(A), *strA, strB, sul2, tet*(A), *gyrA* S83L
15-AB01045_0	*E.coli*	Calf	Caecum	≤ 1	*-*	*-*	ST-410	FII, FIB(AP001918), FIC(FII), I1, Y, Col(BS512), ColRNAI	*aadA1, aph(3')-Ia, bla*_TEM-1_, *dfrA1, floR, strA, strB, sul2, tet*(B), *ampC* T32A, *gyrA* S83L, *gyrA* D87N, *parC* S80I, *parE* S458A
16-AB00129_0	*E.coli*	Pig	Caecum	≤ 1	*-*	*-*	ST-10	FII, FIA, FIB(AP001918), I1, Col(BS512), Col8282	*aadA1, aadA5, bla*_CTX-M-1_, *dfrA17, mph*(A), *strA, strB, sul1, sul2, tet*(A)
16-AB00148_0	*E.coli*	Calf	Caecum	≤ 1	*-*	*-*	ST-349	FII(pCoo), Y	*aac(3)-IVa, aph(4)-Ia, bla*_CTX-M-1_, *mph*(A), *strA, strB*
16-AB00307_0	*E.coli*	Calf	Caecum	≤ 1	*-*	*-*	ST-224	FII, FII(pRSB107), FIA, FIB(AP001918), X1, p0111	*aac(3)-IIa, aac(6')-Ib-cr, aadA5, bla*_CTX-M-15_, *bla*_OXA-1_, *bla*_TEM-1B_, *catB4, dfrA17, mph*(A), *qnrS1, sul1, tet*(A), *gyrA* S83L, *gyrA* D87N, *parC* S80I, *parE* S458A
16-AB00409_0	*E.coli*	Calf	Caecum	≤ 1	*-*	*-*	ST-118	FII(pSE11), FIB(AP001918), B/O/K/Z, I1, Q1	*bla*_CTX-M-1_, *mph*(A), *strA, strB, sul2, tet*(A)
16-AB00430_0	*E.coli*	Calf	Caecum	8	*mcr-1*	*mcr-1*	ST-950	FII, FIB(AP001918), B/O/K/Z, HI2, HI2A, I2, TrfA	*aac(3)-IVa, aadA1, aph(4)-Ia, bla*_CTX-M-1_, *bla*_OXA-1_, *floR, mph*(A), *strA, strB, sul1, sul2, tet*(Y), *gyrA* S83L, *gyrA* D87Y, *parC* S80I
15-SA02327_0	*Salmonella *1,4 [4],12:i:-	Pig	Carcass	≤ 1	*-*	*-*	ST-34	FIB(AP001918), FIC(FII)	*aadA1, aadA2, bla*_TEM-1B_, *cmlA1, dfrA12, mef*(B), *sul3, tet*(B)
**France (n=10)**									
15F001188	*E. coli*	Pig	Animal	≤ 1	*-*	*-*	ST-744	FII, FII(pHN7A8), FIB(AP001918), Q1, Col(MG828), Col156	*aadA5, aph(3')-Ia, bla*_CTX-M-55_, *bla*_TEM-1B_, *dfrA17, fosA3, mph*(A), *strA, strB, sul1, sul2, tet*(B), *gyrA* S83L, *gyrA* D87N, *parC* A56T, *parC* S80I
15F001211	*E. coli*	Calf	Animal	4	*mcr-3*	*mcr-3.2*	ST-744	FIB(AP001918), FIC(FII), Q1	*aac(3)-IId, aadA5, bla*_CTX-M-55_, *bla*_TEM-1B_, *catA1, dfrA17, floR, mph*(A), *strA, strB, sul1, sul2, tet*(B), *gyrA* S83L, *gyrA* D87N, *parC* A56T, *parC* S80I
15F001279	*E. coli*	Calf	Animal	4	*mcr-1*	*mcr-1*	ST-648	FIB(AP001918), FIC(FII), HI2, HI2A, Q1, X1, TrfA, ColRNAI	*aac(3)-IVa, aadA1, aadA2, aph(3')-Ia, aph(4)-Ia, blaTEM-1A, dfrA1, dfrA12, erm*(B), *mph*(B), *strA, strB, sul2, tet*(A), *gyrA* S83L, *gyrA* D87N, *parC* S80I, *parE* L416F
15F002387	*E. coli*	Calf	Animal	4	*mcr-1*	*mcr-1*	ST-744	FIB(AP001918), FIC(FII), HI2, HI2A, Q1, TrfA	*aadA1, aadA2, aadA5, bla*_CTX-M-1_, *bla*_TEM-1B_, *cmlA1, dfrA1, dfrA12, dfrA17, floR, mph*(A), *strA, strB, sul1, sul2, sul3, tet*(A), *tet*(B), *tet*(M), *gyrA* S83L, *gyrA* D87N, *parC* A56T, *parC* S80I
15Q003557	*Salmonella* Rissen	Calf	Carcass	≤ 1	*-*	*-*	ST-469	Col(MG828), ColRNAI	*tet*(A)
15Q003582	*Salmonella* Rissen	Pig	Carcass	≤ 1	*-*	*-*	ST-469	I1, ColRNAI	*aadA1, aadA2, cmlA1, dfrA12, tet*(A), *sul3*
15Q003631	*Salmonella* 4,12:i:-	Pig	Carcass	8	*mcr-1*	*mcr-1*	ST-34	HI2, HI2A, I1, Q1, TrfA, Col156	*aac(3)-IVa, aph(3')-Ia, aph(4)-Ia, bla*_TEM-1B_, *catA1, strA, strB, sul2, tet*(A), *tet*(B)
15Q004074	*Salmonella* 4,12:i:-	Calf	Carcass	4	*mcr-4*	*mcr-4.2*	ST-34	Q1, Col156, ColE10, ColRNAI,	*blaTEM-1B, strA, strB, sul2, tet*(B)
15F001226	*E. coli*	Calf	Animal	≤ 1	*-*	*-*	ST-1011	Q1	*aac(3)-IId, aadA2, bla*_CTX-M-32_, *bla*_TEM-1B_, *catA1, dfrA12, mph*(A), *strA, strB, sul1, sul2, tet*(A), *gyrA* S83L, *gyrA* D87N, *parC* S80I
16F000284	*E. coli*	Pig	Animal	≤ 1	*-*	*-*	ST-871	Col(BS512), ColRNAI, Y	*-*
**Italy (n=4)**									
15051805COYF25	*E. coli*	Calf	Animal	8	*mcr-1*	*mcr-1*	ST-4096	FII, HI2, HI2A, TrfA, ColE10	*aac(3)-IIa, aadA1, aph(3')-Ic, bla*_CTX-M-15_, *dfrA1, floR, strA, strB, sul3, tet*(A), *tet*(M), *gyrA* S83L
150542127ARP05	*E. coli*	Calf	Animal	4	*mcr-1* and *mcr-3*	*mcr-1* and *mcr-3.2*	ST-744	FIB(AP001918), FIC(FII), R, X1, X4, Col156	*aac(3)-IId, aadA2, aadA5, bla*_CTX-M-55_, *bla*_TEM-1B_, *dfrA12, floR, mph*(A), *sul1, sul3, tet*(B), *tet*(M), *gyrA* S83L, *gyrA* D87N, *parC* S80I, *parC* A56T
15056414J9PUD1	*E. coli*	Pig	Animal	4	*mcr-1*	*mcr-1.xx*	ST-5995	FIB(AP001918), FIC(FII)	*catA1*
15058525J9PUD5	*E. coli*	Pig	Animal	≤ 1	*-*	*-*	ST-1684	FIA, FIB(AP001918), FIC(FII), I1, I2, Col(MG828), Col8282, ColRNAI	*aadA1, bla*_CMY-2_, *erm*(C), *tet*(B)

MIC: minimum inhibitory concentration; MLST: multilocus sequence typing; ST: sequence type; WGS: whole genome sequencing.

-: Not detected.

Grey: phenotypic resistance according to the European Committee on Antimicrobial Susceptibility Testing (EUCAST) epidemiological cut-off value [29].

^a^ As determined by using online tools at the Center for Genomic Epidemiology (http://www.genomicepidemiology.org/).

^b^ New variant detected in this study.

### Validation test and comparison with WGS data

The validation test set revealed eight colistin-resistant *E. coli* isolates (MIC 4 – 8 mg/L), five from bovine and three from porcine origin, harbouring the *mcr-1* gene or one of its variants. An additional *E. coli* isolate from a pig phenotypically classified as borderline susceptible (colistin MIC of 2 mg/L), on repeated testing at two laboratories also harboured the *mcr-1* gene. The multiplex PCR also yielded *mcr-1* in one porcine *Salmonella* spp. isolate with colistin MIC of 8 mg/L. One *E. coli* isolate of bovine origin with MIC of 4 mg/L harboured the *mcr-3* gene. Three isolates, with colistin MIC of 4 mg/L, harboured the *mcr-4* gene, corresponding to two *Salmonella* spp. (one isolated from pig and the other from calf) and one porcine *E. coli*. Co-occurrence of *mcr-1* and *mcr-3* was observed in two bovine *E. coli* isolates with colistin MICs of 4 and 8 mg/L, respectively. One porcine *E. coli* isolate with colistin MIC of 8 mg/L harboured both *mcr-1* and *mcr-4* genes.

The results were 100% concordant with WGS data and notably, the multiplex PCR was able to detect a novel variant described in this study for the first time, *mcr-4.3,* that varies in one amino-acid from the original *mcr-4* encoded protein (Table 2). The genome of the isolate harbouring *mcr-4.3* (*Salmonella* *enterica* serovar Kedougou 151570) is deposited in ENA with the accession number ERS1801979. Additionally, the multiplex PCR also detected a *mcr-4* gene with a deletion in nt 441 that alters the reading frame and introduces a premature stop codon. There was one *E. coli* isolate presenting phenotypic resistance to colistin (MIC = 8 mg/L) not due to the presence of *mcr* genes, but instead attributed to the mutations *pmrB* D283G/Y358N in the *pmrA/pmrB* two-component system as determined by analysis of WGS data (Table 2).

## Discussion

The multiplex PCR reaction showed 100% specificity (no unpredicted amplification products) and 100% sensitivity (all of the predicted products were visualised in agarose gel) for the validation set, according to WGS data.

One notable finding was the detection of *mcr-1* in one *E. coli* isolate classified as wildtype (susceptible) (colistin MIC of 2 mg/L). Preliminary results from other studies revealed similar findings of *E. coli* isolates, particularly from poultry, with MIC of 2 mg/L and carrying the *mcr-1* gene, collected in Denmark (data not shown) and Poland (Dariusz Wasyl, National Veterinary Research Institute, Puławy, Poland, personal communication, January 2018). These findings seem to indicate that there is a need to review of the established epidemiological cut-off value for colistin. Also based on the discovery of a new *mcr-4* variant (*mcr-4.3*), it becomes clear that colistin resistance is not yet fully uncovered although our knowledge is evolving rapidly, filling the gaps that compromise the prevention and control of multidrug-resistant bacteria.

Another observation of interest was the detection of two *pmrA*/*pmrB* point mutations in one colistin-resistant isolate, which reveals a need to further investigate and characterise this two-component system in order to accurately predict which mutations lead to phenotypic colistin resistance. Taking into account that the mutations found in this isolate are not consistently associated with a resistant phenotype we cannot confirm, without further analysis, if they represent the underlying mechanism of colistin resistance for this isolate [27,28].

Although the presented multiplex PCR does not allow identification of the specific variants and detects non-functional genes, it still provides valuable information for surveillance purposes as well as for research. The results of the multiplex PCR also allow the selection of isolates for further studies based on genomic epidemiology, focusing on characterisation of *mcr* genes, their mobilisation mechanisms and the plasmids they are harboured on. While the multiplex PCR has not yet been validated on human strains, it is expected to provide the same sensitivity and specificity as for animal isolates. The vast majority of *mcr* genes are located on transferable plasmids that are not restricted to reservoirs or hosts. Similarly, we expect that this multiplex PCR design will maintain its usefulness should new gene variants among *mcr*-*1* to *mcr*-*5* be detected in *Enterobacteriaceae*, taking into account that the chance of variations in the primer annealing positions should be small and no such variations have been observed so far. A great advantage of this method is the ability to detect *mcr* genes in phenotypically susceptible bacteria, allowing it to be used as a screening technique for isolates recovered from human and animal sources. We believe future efforts should include the screening of large pools of borderline susceptible *Enterobacteriaceae* in order to understand if, and how, the epidemiological cut-off value for colistin should be reviewed.

Although an internal amplification control was not used in this study, it should be possible to use a different reaction mix containing another DNA polymerase in order to allow the addition of 16S rRNA primers to obtain an internal reaction control. We encourage researchers to adapt the PCR reaction to their specific needs.

This screening method can provide a useful and accurate description of colistin resistance in *Enterobacteriaceae* collections, particularly when used in connection with MIC susceptibility testing. The protocol and standard operating procedure are freely available at the EURL-AR website (https://www.eurl-ar.eu/). The intended use for this multiplex PCR is as part of the surveillance workflow, to screen colistin-resistant and borderline susceptible isolates after initial MIC determination by the standard broth dilution method. Colistin MIC determination allows classification of isolates as susceptible, borderline susceptible or resistant and, subsequently, the isolates determined borderline susceptible and resistant (MIC ≥ 2 mg/L) can be subjected to the multiplex PCR to provide further insight into the underlying resistance mechanism and potentially reveal the presence of the known *mcr* genes. For an initial surveillance analysis we do not recommend to perform the multiplex PCR before MIC determination, since it could lead to overlooking colistin resistant isolates with other underlying mechanisms of resistance. The method can, however, be used on its own if the research is focused, for example, on plasmid dissemination or *mcr* genes’ epidemiology. Advanced investigations could be conducted according to available procedures and surveillance/research goals, and would ideally involve WGS of the bacterial genome, even though it may not be easily accessible in some laboratories or countries, in particular, low and middle income countries (LMIC). Colistin resistant bacteria lacking any of the currently known *mcr* genes should be screened for other mechanisms of resistance, such as mutations in the *pmrA/pmrB* system, new *mcr* genes or variants, or even presently unknown mechanisms.

Future prospects include validation of the multiplex PCR for human clinical strains, to assure that the protocol follows the principles of the One Health perspective and can be used to directly screen human, veterinary and food bacteria. Other prospects are to clone *mcr-1, mcr-2, mcr-3* and *mcr-5* amplicons into TA-cloning vectors (Thermo Fisher Scientific) in order to obtain non-pathogenic and easy to handle control strains, and be able to confirm and update the protocol continuously as new variants are being detected.

In conclusion, we developed a multiplex PCR that rapidly detects all *mcr* genes described in *Enterobacteriaceae* to date. The multiplex PCR method is critical for epidemiological surveillance and antimicrobial resistance investigation, especially in settings where laboratory resources and access to WGS are limited, and where plasmid-mediated colistin resistance may threaten the effectiveness of available antimicrobials.

## * Erratum

Due to a technical error, the name of Cristina De Frutos Escobar was incorrectly displayed in the list of authors. The mistake was corrected on 12 Feb 2018.
