# Erratum for Euro Surveill. 2018;23(6)

**DOI:** 10.2807/1560-7917.ES.2018.23.7.180215-1

**Published:** 2018-02-15

**Authors:** Eurosurveillance editorial team

**Affiliations:** 1 European Centre for Disease Prevention and Control (ECDC), Stockholm, Sweden

In the article ‘Multiplex PCR for detection of plasmid-mediated colistin resistance determinants, *mcr-1*, *mcr-2*, *mcr-3*, *mcr-4* and *mcr-5* for surveillance purposes’, published on 8 February 2018, the name of Cristina De Frutos Escobar was incorrectly displayed in the list of authors. The mistake was corrected on 12 Feb 2018.

